# Intraoperative mobile CT and virtual-assisted lung mapping for pulmonary nodule localization: workflow characteristics and radiation dosage in a single-center experience

**DOI:** 10.1007/s11748-026-02342-3

**Published:** 2026-06-25

**Authors:** Keita Nakao, Takahiro Karasaki, Yue Cong, Takaki Akamine, Masahiro Yanagiya, Masaaki Nagano, Mitsuaki Kawashima, Gouji Toyokawa, Chihiro Konoeda, Masaaki Sato

**Affiliations:** 1 Department of Thoracic Surgery, The University of Tokyo Graduate School of Medicine, 7-3-1 Hongo, Bunkyo-ku 113-8655 Tokyo, Japan; 2 Department of Thoracic Surgery, Japanese Red Cross Omori Hospital, 4-30-1 Chuo, Ota-ku 143-8527 Tokyo, Japan; 3 Department of Thoracic Surgery, Kyushu University Hospital, 3-1-1 Maidashi, Higashi-ku, Fukuoka-shi 812-8582 Fukuoka, Japan; 4 Department of Thoracic Surgery, Japan Institute for Health Security, 1-21-1 Toyama, Shinjuku-ku 162-865 Tokyo, Japan

**Keywords:** Pulmonary nodules, Thoracic surgery, Video-assisted, Computed tomography, Intraoperative imaging, Virtual-assisted lung mapping (VAL-MAP)

## Abstract

**Objectives:**

Intraoperative mobile CT (iCT) and virtual-assisted lung mapping (VAL-MAP) are used to localize pulmonary nodules that are difficult to palpate during minimally invasive surgery. Because these techniques differ in workflow structure and timing of image acquisition, their operative time and radiation exposure may differ. We aimed to describe workflow characteristics and radiation exposure associated with newly implemented iCT and to contextualize these findings against our established VAL-MAP practice.

**Methods:**

We retrospectively reviewed 50 consecutive patients who underwent thoracoscopic wedge resection with localization between January 2024 and December 2025. Twenty-four underwent iCT-guided localization using an O-arm system, and 26 underwent VAL-MAP with bronchoscopic dye marking followed by post-mapping CT. Technique selection was based on device availability and surgeon discretion. CT dose metrics and time components related to localization were analyzed.

**Results:**

Median CT-related dose-length product (DLP) was higher in the iCT group, primarily due to high-definition scans. When performed in standard mode with a limited scan frequency, iCT radiation exposure approximated that of post-VAL-MAP CT. Preparation CT after lateral positioning reduced repeat scans and iCT-related interruption time. Anesthesia and operative times were shorter in the VAL-MAP cohort, reflecting localization outside the operating room. Overall procedural burden appeared modestly different.

**Conclusion:**

Our single-center retrospective study quantified the total procedural time for VAL-MAP and iCT, finding modest differences in time and radiation exposure. These real-world data aid institutions considering these localization strategies, but do not establish superiority. Definitive comparative conclusions require future prospective evaluations controlling for nodule characteristics and cost.

**Supplementary Information:**

The online version contains supplementary material available at https://doi.org/10.1007/s11748-026-02342-3.

## Introduction

Surgical resection of pulmonary nodules that lack pleural changes and that are difficult to palpate remains a considerable challenge for thoracic surgeons, and such difficulty is amplified in the era of minimally invasive thoracoscopic and robot-assisted surgery. Localization and visualization of an impalpable lung nodule can be performed preoperatively or intraoperatively [1]. Preoperative computed tomography (CT)-guided hook wire injection is used; however, due to the rare but severe risk of air embolism [2], alternative methods have been developed. One such preoperative localization method is virtual-assisted lung mapping (VAL-MAP) [3–6]. Others have developed the placement of radiofrequency identification tags under bronchoscopy, which is also a promising approach that can be implemented in a wide range of institutions, as it requires only the purchase of equipment and no major construction [7, 8], or the application of endobronchial dye marking guided by cone-beam CT-derived augmented fluoroscopy [9].

Advancements in CT technology have also facilitated the increasing availability of intraoperative CT imaging [10–15]. Metal clips are applied to the visceral pleura, and the spatial relationship against the nodule can be visualized using an intraoperative mobile CT scan. Localization of the nodule can also be supported by CT-guided skin marking after the patient is positioned on the operating table.

Standard bronchoscopy skills and equipment, a bronchoscopy infusion catheter, and 3D-CT reconstruction software are the requisites for VAL-MAP implementation, all of which can be equipped in a hospital providing standard-of-care lung cancer treatment [3]. Intraoperative mobile CT (excluding those that require a hybrid operating room [OR] and CT-on-rail system) could also be implemented without renovation of the OR. Therefore, these two localization techniques, VAL-MAP and intraoperative mobile CT, could be implemented in a broad range of institutions. In our institution, we discontinued the application of the CT-guided hookwire injection method, and VAL-MAP was implemented in 2014. Intraoperative cone-beam mobile CT was later implemented in 2024.

These techniques permit accurate localization of nodules that are difficult to palpate or visualize, thereby enabling appropriately tailored lung resection, at the expense of additional operative time and radiation exposure. However, the extent of the additional operative time and radiation exposure associated with each technique remains unclear.

In this study, we report our initial experience with intraoperative CT (iCT) and its workflow characteristics, and assess the additional operative time and radiation exposure between iCT and VAL-MAP. Importantly, our objective is not to determine the superiority of one method over the other, as the choice of technique often relies on institutional expertise, specific patient conditions (e.g., lack of consent for bronchoscopy, contrast allergy), and surgeon preference. Instead, by contextualizing the real-world data of newly implemented iCT against an established VAL-MAP practice, we aim to provide practical insights for protocol optimization and workflow management for institutions considering the introduction of alternative pulmonary nodule localization techniques.

## Materials and methods

### Study design and patients

We retrospectively reviewed cases of thoracoscopic wedge resection for pulmonary nodules, with iCT or VAL-MAP performed, at The University of Tokyo Hospital from January 2024 to December 2025. During the study period, no other localization techniques were applied. Only cases with a single target lesion were included. The decision to use a lesion localization method and the choice of specific technique were based on the availability of iCT and the operating surgeon’s discretion, without predefined allocation criteria. All procedures were discussed at an intra-departmental conference prior to admission for the surgery.

### iCT technique

Intraoperative CT was performed using the O-arm™ imaging system (Medtronic, Minneapolis, MN, USA) in combination with a radiolucent tabletop from the Trios Table System™ (Mizuho, Tokyo, Japan). The patient underwent general anesthesia with a double-lumen tube and was positioned in the lateral recumbent position for the surgery. Patient’s both arms were carefully adjusted to prevent interference with the CT. Preparation CT was performed after the patient was positioned in the recumbent position to confirm the position of the mobile CT and, in most cases, to perform cutaneous marking; however, this step was omitted in some cases. The position of the preparation CT was marked on the floor, and the optimized height of the surgical table was recorded to facilitate safe, easy gantry repositioning during surgery.

The surgery was performed via a three-port thoracoscopic approach. After port placement and inspection of the thoracic cavity, visceral pleural marking was performed using surgical metal clips with or without a guide of visceral pleural dye marking that was applied through the following steps. 2-0 Nylon suture string was delivered into the thoracic cavity via the intravenous catheter inserted through the chest wall at the cutaneous marking. The suture string was then used to affix the gauze ball impregnated with indigo carmine dye to the parietal pleura. By completely inflating the lung, the visceral pleura adjacent to the tumor could be marked with indigo carmine. After the surgical clips were applied to the visceral pleura, the O-arm was introduced into the operative field. After confirming the spatial relationship between the tumor and each metal clip on the acquired CT images, the O-arm was rolled out, and a pulmonary wedge resection was performed using a surgical stapler.

O-arm imaging was performed according to the default thoracic spine radiation-dose protocol, with either the standard (120 kV, 125 mAs) or high-definition (120 kV, 186 mAs) dose setting, as previously described [16]. Body size was set as S for all cases. The default range of the 3D field-of-view had a diameter of 20 cm and a length of 15 cm. Two-dimensional (2D) fluoroscopic images were acquired to verify the gantry position; this step was optional and could be omitted, particularly when the preoperative preparation CT was obtained after the patient was placed in the lateral decubitus position.

In our institution, thoracic surgeons manipulate the O-arm, but radiology technicians can also do so, requiring a minimum of one additional surgeon or technician in the OR during the procedure.

### VAL-MAP technique

The detailed procedure for VAL-MAP is described in the Supplemental Document S1. Briefly, a bronchoscopic dye marking guided by virtual bronchoscopy images was performed prior to surgery. Following the bronchoscopy, a CT scan (post-VAL-MAP CT) was acquired to create a three-dimensional (3D) map showing the spatial relationship between the target lesion and the dyes. In our institution, a minimum of 2 thoracic surgeons perform bronchoscopic procedures for VAL-MAP, but these procedures can also be performed by pulmonary physicians, requiring a minimum of 2 surgeons or physicians outside the OR. Post-VAL-MAP CT scanning and 3D reconstruction are performed by a radiology technician. Markings were confirmed using both white-light and near-infrared imaging during surgery. Following confirmation of the markings, a pulmonary wedge resection was performed using a surgical stapler via a complete thoracoscopic approach.

### Data collection and analysis

Clinical data were retrospectively collected from electronic medical records. Operative video records were reviewed to measure the time spent on intraoperative marking and CT scanning.

All statistical analyses and data visualization were performed using R (v.3.6.1; R Foundation for Statistical Computing, Vienna, Austria). For comparisons between the two groups, Fisher’s exact test was used for categorical variables, while Wilcoxon’s rank-sum test, Kruskal-Wallis, and Steel-Dwass tests were used for continuous variables. Continuous data were presented as median and interquartile range (IQR), and categorical data were presented as counts. A two-tailed p-value of < 0.05 was considered statistically significant.

## Results

### Characteristics of patients

A total of 50 patients were included in this study: 24 who underwent localization with intraoperative CT (iCT group) and 26 who underwent localization with VAL-MAP (VAL-MAP group). The baseline patient characteristics are summarized in Table 1 and Supplementary Table S2. The number of applied markings was significantly fewer in the iCT group (fraction of 2 or fewer markings; iCT group, 10/24; VAL-MAP group, 2/26; *p* = 0.0074). The majority of patients in the iCT group underwent CT scanning twice (16/24) in the operating room under the standard-dose mode (13/24). All target nodules had been successfully resected with a macroscopically clear margin (typically defined as a margin distance greater than the tumor diameter or at least 2 cm), and no additional resection was performed. No complication related to bronchoscopic procedure was observed in the VAL-MAP group. There was no difference in intraoperative blood loss (median < 10 ml in both groups), the duration of chest tube insertion (median 1 day in both groups), or the length of hospital stay after surgery between the two groups (iCT, median 6 [IQR 4.75, 6] days; VAL-MAP, median 5 [IQR 4, 6] days; *p* = 0.68). There was no difference in the CT appearance of the nodules (fraction of pure or part-solid GGN; iCT, 10/24; VAL-MAP, 14/26; *p* = 0.41), total tumor size in the CT image (iCT, median 8 [IQR 6, 9.25] mm; VAL-MAP, 9 [7, 12.75] mm; *p* = 0.39), and the depth from visceral pleura in the CT (iCT, median 4.5 [IQR 2.5, 14.5] mm; VAL-MAP, 3.5 [1.25, 7] mm; *p* = 0.14) between the two groups. It is of note that, however, more benign nodules were resected using iCT compared with VAL-MAP during the study period (iCT, 9/24; VAL-MAP, 0/26; *p* = 0.00052). These results suggest that surgeons may have chosen iCT rather than VAL-MAP for more indeterminate nodules, and any comparison between the two methods might be affected by such selection biases.

**Table 1:** Patient characteristics

	VAL-MAP	Intraoperative CT
	(*N* = 26)	(*N* = 24)
Sex		
Male	13	10
Female	13	14
Age, median (years)	67.5	66
(IQR)	(54, 76)	(58,72)
Number of markings		
1	0	3
2	2	7
3	17	12
4	7	2
Number of CT scanning		
1	26	3
2	0	16
3	0	5
Mobile CT settings		
Standard mode	-	13
High-definition mode	-	10
Both	-	1
Pathological diagnosis		
Malignant	26	15
Benign	0	9
Chest tube insertion, median (days)	1	1
(IQR)	(1,2)	(1,2)
Postoperative hospital stay, median (days)	5	6
(IQR)	(4,6)	(4.75,6)

IQR, interquartile range

### Comparison of radiation dose by CT scans between iCT and VAL-MAP

Radiation dose associated with localization procedures was compared between the iCT and VAL-MAP groups. The median radiation dose exposed during the VAL-MAP marking procedure using bronchoscopy was 2.57 mGy (IQR 1.94, 3.55), while the median CT dose index of post-VAL-MAP CT was 8.17 mGy (6.80, 10.02). Although it is difficult to directly compare or combine radiation doses between 2D and 3D settings, given the higher radiation dose of CT compared with fluoroscopy during bronchoscopy, we reasoned that the comparison of radiation doses between VAL-MAP and iCT could focus on the radiation dose during the CT scan. Detailed CT metrics per case, including the iCT (O-arm imaging system) scan mode, number of scans in the OR, and DLP, are provided in Supplemental Table S3.

**Fig. 1:**
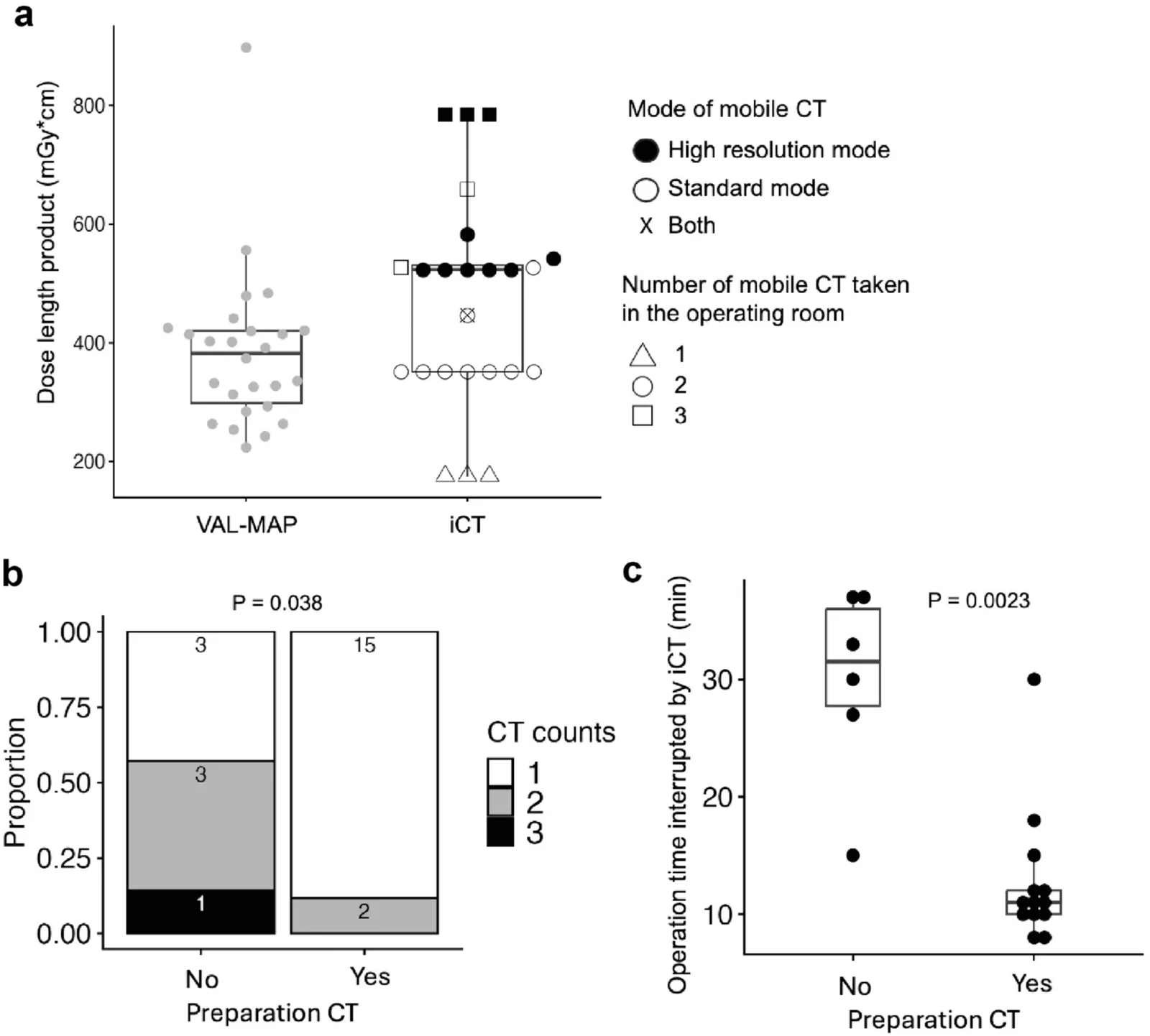
Assessment of the CT scan-related metrics. **a** Assessment of radiation dose length product (mGy*cm) between VAL-MAP and intraoperative CT (iCT) groups. Black and white marks denote high-definition and standard modes in iCT settings, respectively. The shape of the marks represents the number of CT scans performed in the iCT group. **b** Barplot showing the relations between the presence of preparation CT and the number of intraoperative CT scans in the iCT group. **c** Distribution of the surgery time interrupted by intraoperative CT

The iCT group demonstrated significantly higher Dose Length Product (DLP) than VAL-MAP (median DLP of iCT, 523.4 [IQR 351.4, 530.9]; VAL-MAP, 382.9 [298.6, 420.4], *p* = 0.032) (Fig. 1a). Such a difference was due to excessively high DLP yield by iCT taken under high-definition mode (median DLP of high-resolution iCT, 532.8 [IQR 523.4, 734.5]; standard mode iCT, 351.4 [351.4, 351.4]; *p* = 0.0026). The data indicated that using iCT in standard mode twice or less, or in both standard and high-definition modes once each, would yield a radiation dose comparable to that of post-VAL-MAP CT.

### Utility of preparation CT in the recumbent position before surgery in the iCT method

Preparation CT was performed to confirm the position of the mobile CT, visualize the nodule, and apply cutaneous markings to guide pleural marking during surgery. This procedure was omitted in 7 cases to reduce the total number of scans. Therefore, we next assessed whether omitting preparation CT could reduce the total number of scans. Three of the 7 cases could be completed with a single scan. However, the rest of 4 cases underwent 2 or more CT scans during the surgery, resulting in a higher rate of multiple scans during surgery compared with the cases that underwent preparation CT before surgery (without preparation CT, 4/7; with preparation CT, 2/17; *p* = 0.038) as well as a longer time of surgery interruption by intraoperative CT (median interruption time, 31.5 [IQR 27.75, 36] minutes vs. 11 [10, 12] minutes; *p* = 0.0023) (Fig. 1b, c). These results suggested that, although omitting preparation CT before surgery could occasionally minimize the number of CT scans, preparation CT could overall reduce the risk of repeat CT scans during surgery.

### Analysis of time consumed for localization procedures

**Fig. 2:**
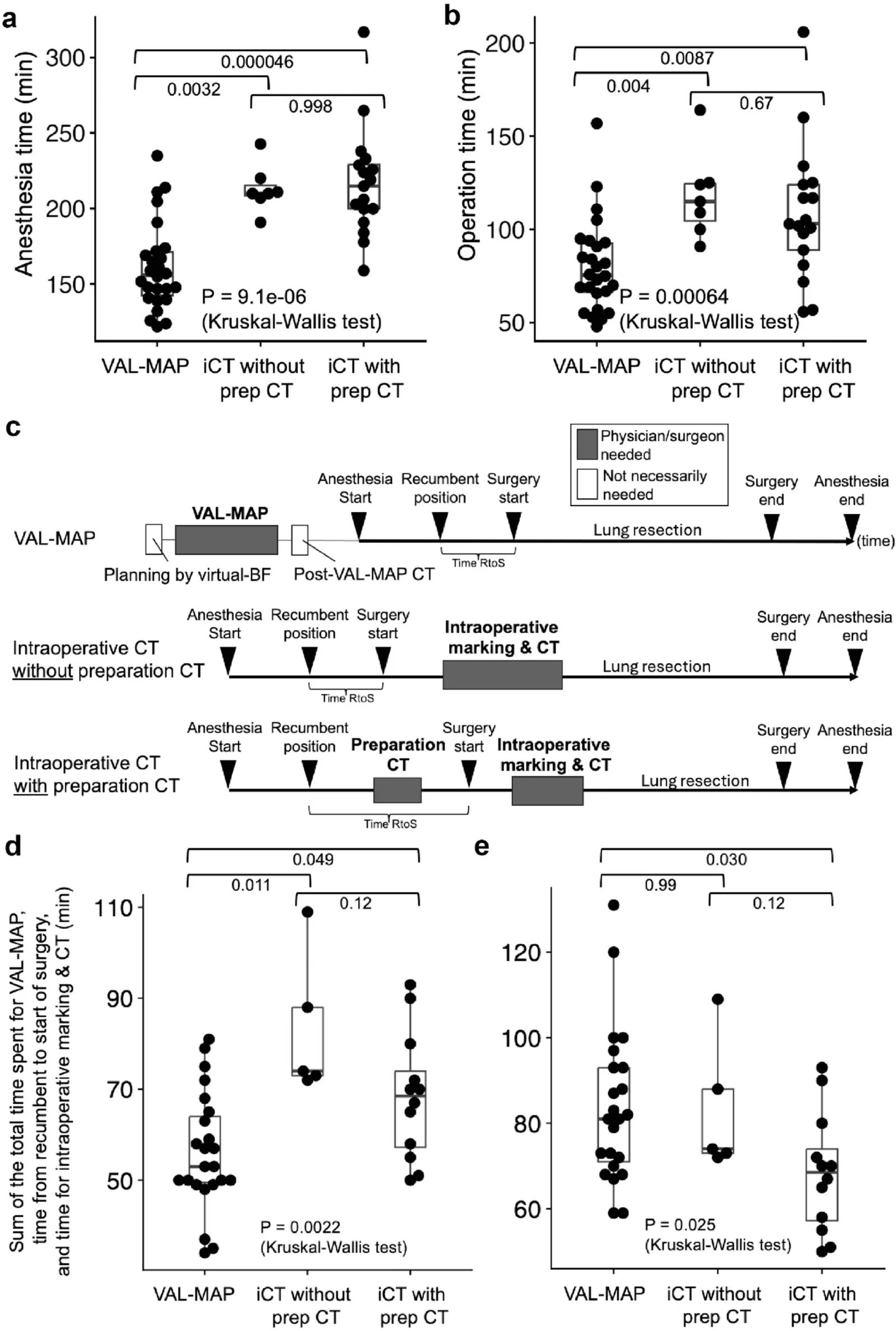
Assessment of procedure time related to localization. **a**, ** b** Anesthesia time **(a)** and operation time **(b)** were compared across three groups: VAL-MAP, intraoperative mobile CT (iCT) without preparation CT (prep CT), and iCT with prep CT. **c** Schematics of time courses based on localization and marking procedures. **d**, ** e** The time for the VAL-MAP procedure (zero for the iCT group), the time from recumbent position to the start of operation, and the time for intraoperative marking and CT (zero for the VAL-MAP group) were calculated, and their sum was compared across localization procedures. Time consumed for VAL-MAP was measured in two ways: the time from airway anesthesia to the end of the bronchoscopic procedure (**d**) and the time from airway anesthesia to the end of post-VAL-MAP CT (**e**). Time RtoS, time from recumbent to start of surgery; iCT, intraoperative CT; prep CT, preparation CT

When the anesthesia and operation times were compared across iCT and VAL-MAP groups, the VAL-MAP group showed significantly shorter anesthesia time (median anesthesia time in VAL-MAP, 156 [IQR 142.5, 171.2] minutes; iCT without preparation CT, 210 [208.5, 215.5]; iCT with preparation CT, 215 [200, 229]; *p* < 0.0001) and operation time (VAL-MAP, 75.5 [66.25, 92.5] minutes; iCT without preparation CT, 115 [104.5, 124.5]; iCT with preparation CT, 103 [89, 124]; *p* = 0.00064) (Fig. 2a, b). These results indicate that VAL-MAP can reduce operating room time for pulmonary nodules that require an additional localization method upon wedge resection compared with iCT. Notably, the omission of preparation CT in the iCT group was not associated with shorter anesthesia or operation times, suggesting that omitting preoperative preparation CT in the iCT method may not reduce overall operating room time.

Lastly, to assess the overall time spent on localization procedures, the time spent on each process was measured and summed. Because we could not directly measure the time spent on the preoperative preparation CT in the iCT group, we instead calculated the duration from the time the patient was fixed in a recumbent position until surgery began (Fig. 2c). The total time for VAL-MAP (bronchoscopic procedural time from airway anesthesia to the end of procedure; zero for the iCT group), the time from recumbent position to the start of operation (which includes the time for preoperative preparation CT in the iCT group), and the time for intraoperative marking and CT (zero for the VAL-MAP group) were measured, and their sum was calculated to grossly assess the time consumed for the localization procedures performed in distinct workflows. When the post-VAL-MAP CT time was not included in the total time for VAL-MAP (i.e., when the total time for VAL-MAP was defined as the time from airway anesthesia to the end of bronchoscopy), the VAL-MAP group showed the shortest time consumed for the localization procedure (*p* = 0.0022) (Fig. 2d). However, when the definition of the end time for VAL-MAP was extended until the time when post-VAL-MAP CT was taken, iCT with preparation CT showed the shortest time consumed for the localization procedure (*p* = 0.025, Kruskal-Wallis test) (Fig. 2e). Neither method of estimating the time consumed for VAL-MAP accounts for the time spent planning VAL-MAP. Overall, these results indicate that the difference in time required for localization procedures appeared modest within this cohort, and that the comparison is strongly biased by how each method’s time is measured.

## Discussion

To offer practical information for institutions considering the implementation of localization techniques for pulmonary nodules difficult to identify during surgery, we presented the workflow characteristics, with a special focus on time and radiation dose, for two techniques that can be implemented across a wide range of hospitals where a standard-of-care treatment for lung cancer is provided: iCT and VAL-MAP. Our goal is not to establish the superiority of one technique over the other, but rather to provide a practical reference for institutions that have not yet adopted any localization method, or those seeking alternative options when their primary method cannot be performed (e.g., due to patient refusal of bronchoscopy or contrast allergy). In this retrospective study, we evaluated the extent of additional operative time and radiation dose associated with localization procedures. VAL-MAP was associated with shorter anesthesia and operative times by allowing localization outside the operating room, whereas iCT enabled all localization steps to be completed intraoperatively. However, iCT was often selected for more indeterminate nodules, suggesting a selection bias. Although we observed slightly higher radiation dose for CT in the iCT group than in the VAL-MAP group, this was driven primarily by scans performed in high-definition mode in the O-arm imaging system. When the O-arm was used for intraoperative mobile CT, two scans in standard mode, or in both standard and high-definition modes, each yielded a radiation dose comparable to that of post-VAL-MAP CT, consistent with a previous report [11]. While the limited sample size and patient selection bias preclude definitive conclusions regarding radiation exposure, our exploratory comparison suggested that the high-definition mode could be a source of excess radiation in the iCT group. This real-world finding helped us optimize our protocol by considering standard mode settings, which may serve as a useful reference for other institutions during their initial implementation phase. Overall, there was no clinically meaningful difference in radiation exposure between the 2 methods, and the exposure in each method was modest, given the cumulative radiation exposure during oncological surveillance before and after surgery. Regarding CT scan radiation dose, the O-arm imaging protocol at our institution is standardized for spinal surgery and may not be optimized for lung imaging. In our experience, the standard dose setting is often satisfactory for visualization of both solid and ground glass nodules (GGN); however, we suggest using a high-definition dose setting until the operating team gets used to the handling of the system and the procedures, and for the small nodules (< 1 cm) with pure GGN appearances. The finding that the preparation CT in the lateral recumbent position before the surgical field was sterilized was associated with fewer CT scans during surgery and shorter surgery time interrupted by CT may be especially useful for institutions planning to implement iCT methods, although the result was from a subset analysis with a small number of patients. Therefore, performing a preparation CT before the surgical field is sterilized may be considered a beneficial step to optimize the iCT workflow and minimize surgical interruption.

Overall, the differences in the total time required for each procedure appeared modest. A potential contribution of this analysis is the quantitative comparison of the overall time burden by aggregating the pre-procedural time spent outside the operating room (e.g., bronchoscopy and post-mapping CT for VAL-MAP) and the intraoperative time spent inside the operating room (e.g., preparation CT and scan interruptions for iCT). By doing so, we demonstrated that the total time burden is comparable between the two approaches. The two localization techniques have distinct advantages: iCT can be applied to patients who are unfit for, or unwilling to undergo, bronchoscopy, and the procedure can be completed in the operating room. VAL-MAP, by contrast, can be performed outside the operating room before surgery and may save anesthesia and operating times.

One limitation of the time analysis in the current study is that we could not assess the time required to plan VAL-MAP using virtual bronchoscopy imaging, which may take approximately 30–60 min, even with software assistance. VAL-MAP planning time was not directly measured and therefore “total burden” of time assessed in the current study should be interpreted cautiously. It is also notable that the VAL-MAP was introduced nearly 10 years ago at our institution, whereas iCT was introduced 2 years ago. Further refinement of the iCT procedure is expected as experience accumulates. We acknowledge that comparing a newly introduced iCT technique with an established VAL-MAP method precludes any definitive evaluation of their comparative efficacy. Rather, our findings should be interpreted as a descriptive analysis of the workflow characteristics during the initial implementation phase of iCT. There are other limitations in the current study. Firstly, because this study was retrospective and not designed as a cost-analysis study, precise cost comparison (including equipment amortization and maintenance contracts) could not be reliably reconstructed. A formal cost-effectiveness analysis remains an important subject for future research. Secondly, other localization techniques, including the RFID method or CT-guided percutaneous dye marking [7, 8, 17], that can also be implemented at most hospitals, are not used in our institution and therefore could not be compared. Thirdly, the radiation dose by 2D fluoroscopy was not considered in the comparison between the groups, as described in the results section. Moreover, the patient’s absorbed radiation dose was not directly measured; it was estimated from the applied dose. In addition, radiation exposure among medical staff was not measured. Fourthly, technique selection was based on device availability and surgeon discretion, without predefined allocation criteria, resulting in a strong selection bias. As noted, nodules in the VAL-MAP group tended to be closer to the pleura, which may have influenced the choice of technique. Any comparison between the groups might be affected by selection bias, and, given the limited sample size, comparative conclusions regarding superiority cannot be drawn. Especially, the subgroup analyses were highly exploratory and require further validation. Lastly, future prospective studies assessing short- and long-term outcomes with detailed assessment of surgical margins, incorporating standardized lesion-level variables to control for selection biases and rigorous cost-effectiveness analyses, are required.

## Conclusion

Workflow characteristics of two localization procedures are presented. By quantifying and aggregating the respective out-of-operating-room and in-operating-room procedural times, we found that the overall time burden and radiation exposure required for each procedure differed only slightly. Rather than demonstrating the superiority of one method, this study highlights that each method serves as a viable alternative depending on institutional circumstances. Furthermore, analyzing our early experience allowed us to identify protocol optimizations. Specifically, when applying the iCT method, a preoperative preparation CT in the lateral recumbent position, performed before the surgical field is sterilized, was associated with fewer CT scans during surgery and shorter surgery time interrupted by CT.

## Supplementary Information

Below is the link to the electronic supplementary material.

Supplementary Material 1

Supplementary Material 2

Supplementary Material 3
