# Supplementary material for: Intraoperative mobile CT and virtual-assisted lung mapping for pulmonary nodule localization: workflow characteristics and radiation dosage in a single-center experience
Source: Gen Thorac Cardiovasc Surg. 2026 Jun 25;74(11):1116–24. doi: 10.1007/s11748-026-02342-3 (PMC13690832; doi:10.1007/s11748-026-02342-3)
Supplement: Supplementary file 1 [file 11748_2026_2342_MOESM1_ESM.docx]

Supplemental Document

**Detailed Virtual-Assisted Lung Mapping (VAL-MAP) Procedure**

In the VAL-MAP procedure, a high-resolution computed tomography (CT) scan is utilized to create a virtual bronchoscopy. Bronchoscopic subpleural dye marking is conducted under fluoroscopic guidance on the day before surgery or in the morning of the day of surgery. The bronchoscopy route was optimized using virtual bronchoscopy to enable precise bronchoscopic marking with the VAL-MAP-DS technique. After the bronchoscopy, a high-resolution CT scan (post-VAL-MAP CT) with a slice thickness of 1 mm or less is performed with the patient in a lateral recumbent position (affected side up). This post-mapping CT is used to create a three-dimensional (3D) map, confirming the positional relationship between the target nodule and the marked spots to guide the surgical resection lines.

For the surgery, the patient underwent general anesthesia with a double-lumen tube and was placed in the lateral decubitus position. The surgical procedure was performed via a complete thoracoscopic approach. Markings were confirmed using both white-light and near-infrared imaging with the VISERA Elite II (Olympus, Tokyo, Japan). Following confirmation of the markings, a pulmonary wedge resection was performed using a surgical stapler.

References

[Sato M, Omasa M, Chen F, Sato T, Sonobe M, Bando T, et al. Use of virtual assisted lung mapping (VAL-MAP), a bronchoscopic multispot dye-marking technique using virtual images, for precise navigation of thoracoscopic sublobar lung resection. J Thorac Cardiovasc Surg. 2014;147: 1813–1819.](http://paperpile.com/b/8MqZ1M/zUGU)

[Nagano M, Sato M. Ten-year outcome and development of virtual-assisted lung mapping in thoracic surgery. Cancers. 2023;15: 1971.](http://paperpile.com/b/8MqZ1M/qglB)

[Sato M. Precise sublobar lung resection for small pulmonary nodules: localization and beyond. Gen Thorac Cardiovasc Surg. 2020;68: 684–691.](http://paperpile.com/b/8MqZ1M/NKdW)

[Yanagiya M, Sato M, Ijiri N, Kobayashi K, Nagano M, Konoeda C, et al. Virtual-assisted lung mapping using dual staining with indocyanine green and indigo carmine enhanced marking detectability. J Thorac Dis. 2022;14: 1061–1069.](http://paperpile.com/b/8MqZ1M/0GfV)
