# Supplementary material for: Intraoperative mobile CT and virtual-assisted lung mapping for pulmonary nodule localization: workflow characteristics and radiation dosage in a single-center experience
Source: Gen Thorac Cardiovasc Surg. 2026 Jun 25;74(11):1116–24. doi: 10.1007/s11748-026-02342-3 (PMC13690832; doi:10.1007/s11748-026-02342-3)
Supplement: Supplementary file 2 [file 11748_2026_2342_MOESM2_ESM.docx]

**Supplementary Table S1. Summary of target nodules.**

|  | VAL-MAP | Intraoperative CT |
| --- | --- | --- |
|  | (N=26) | (N=24) |
| Nodule type |  |  |
| Pure GGN | 12 | 7 |
| Part-solid GGN | 2 | 3 |
| Solid | 12 | 14 |
| CT total size, median (mm) | 9 | 8 |
| (IQR) | (7,12.75) | (6,9.25) |
| CT depth, median (mm) | 3.5 | 4.5 |
| (IQR) | (1.25,7) | (2.5,14.5) |
| Pathological diagnosis |  |  |
| Primary lung cancer | 16 | 9 |
| Pulmonary metastasis | 10 | 6 |
| Atypical adenomatous hyperplasia | 0 | 3 |
| Ciliated muconodular papillary tumor | 0 | 2 |
| Hamartoma | 0 | 1 |
| Inflammatory | 0 | 3 |
|  |  |  |

IQR, interquartile range
