# Supplementary material for: Intraoperative mobile CT and virtual-assisted lung mapping for pulmonary nodule localization: workflow characteristics and radiation dosage in a single-center experience
Source: Gen Thorac Cardiovasc Surg. 2026 Jun 25;74(11):1116–24. doi: 10.1007/s11748-026-02342-3 (PMC13690832; doi:10.1007/s11748-026-02342-3)
Supplement: Supplementary file 3 [file 11748_2026_2342_MOESM3_ESM.docx]

**Supplementary Table S2. Detailed CT metrics per case.**

| Group | Preparation CT at recumbent position in OR | Number of CT scan in OR | Total DLP (mGycm) | O-arm CT settings  (mode & diameter) |
| --- | --- | --- | --- | --- |
| iCT | No | 1 | 175.7 | standard 20cm |
| iCT | No | 2 | 446.66 | standard 20cm &  high definition 40cm |
| iCT | No | 1 | 175.7 | standard 20cm |
| iCT | No | 1 | 175.7 | standard 20cm |
| iCT | No | 2 | 351.4 | standard 20cm |
| iCT | No | 2 | 527.09 | standard 20cm |
| iCT | No | 3 | 659.24 | standard 40cm |
| iCT | Yes | 2 | 351.4 | standard 20cm |
| iCT | Yes | 3 | 527.09 | standard 20cm |
| iCT | Yes | 2 | 351.4 | standard 20cm |
| iCT | Yes | 2 | 351.4 | standard 20cm |
| iCT | Yes | 2 | 351.4 | standard 20cm |
| iCT | Yes | 3 | 785.06 | high definition 20cm |
| iCT | Yes | 2 | 523.38 | high definition 20cm |
| iCT | Yes | 2 | 523.38 | high definition 20cm |
| iCT | Yes | 2 | 523.38 | high definition 20cm |
| iCT | Yes | 2 | 523.38 | high definition 20cm |
| iCT | Yes | 2 | 351.4 | standard 20cm |
| iCT | Yes | 2 | 583.01 | high definition (40cm & 20cm) |
| iCT | Yes | 2 | 542.28 | high definition 40cm |
| iCT | Yes | 2 | 523.38 | high definition 20cm |
| iCT | Yes | 3 | 785.06 | high definition 20cm |
| iCT | Yes | 3 | 785.06 | high definition 20cm |
| iCT | Yes | 2 | 351.4 | standard 20cm |
| VAL-MAP | - | 0 | 556.02 | - |
| VAL-MAP | - | 0 | 441.99 | - |
| VAL-MAP | - | 0 | 401.5 | - |
| VAL-MAP | - | 0 | 403.02 | - |
| VAL-MAP | - | 0 | 484.26 | - |
| VAL-MAP | - | 0 | 373.95 | - |
| VAL-MAP | - | 0 | 332.18 | - |
| VAL-MAP | - | 0 | 391.87 | - |
| VAL-MAP | - | 0 | 253.48 | - |
| VAL-MAP | - | 0 | 263.36 | - |
| VAL-MAP | - | 0 | 223.82 | - |
| VAL-MAP | - | 0 | 420.66 | - |
| VAL-MAP | - | 0 | 284.32 | - |
| VAL-MAP | - | 0 | 326.42 | - |
| VAL-MAP | - | 0 | 328.02 | - |
| VAL-MAP | - | 0 | 425.33 | - |
| VAL-MAP | - | 0 | 242.82 | - |
| VAL-MAP | - | 0 | 414.97 | - |
| VAL-MAP | - | 0 | 336.35 | - |
| VAL-MAP | - | 0 | 293.71 | - |
| VAL-MAP | - | 0 | 419.8 | - |
| VAL-MAP | - | 0 | 263.8 | - |
| VAL-MAP | - | 0 | 897.53 | - |
| VAL-MAP | - | 0 | 414.55 | - |
| VAL-MAP | - | 0 | 313.22 | - |
| VAL-MAP | - | 0 | 480.02 | - |

OR, operating room; DLP, dose length product
